# Double-hit lymphoma of the male breast: a case report

**DOI:** 10.1186/s13256-020-02526-2

**Published:** 2020-12-18

**Authors:** Shaymaa Elgaafary, Inga Nagel, Cristina López, Susanne Bens, Monika Szczepanowski, Rabea Wagener, Wolfram Klapper, Reiner Siebert

**Affiliations:** 1 Institute of Human Genetics, Ulm University and Ulm University Medical Center, D-89081 Ulm, Germany; 2 Institute of Human Genetics, Christian-Albrechts University Kiel and University Hospital Schleswig-Holstein, Campus Kiel, D-24105 Kiel, Germany; 3 Division of Human Genetics and Genome Research, Department of Human Cytogenetic, Cairo, 12622 Egypt; 4 Institute of Experimental and Clinical Pharmacology, Christian-Albrechts University Kiel and University Hospital Schleswig-Holstein Campus Kiel, D-24105 Kiel, Germany; 5 Department of Internal Medicine II (Hematology), Laboratory of Hematology, University Hospital, Schleswig-Holstein Campus Kiel, D-24105 Kiel, Germany; 6 Hematopathology Section and Lymph Node Registry, Institute of Pathology Christian-Albrechts University Kiel and University Hospital Schleswig-Holstein, Campus Kiel, D-24105 Kiel, Germany

**Keywords:** Lymphoma, *MYC*, *BCL2*, breast, male, Burkitt

## Abstract

**Background:**

Whereas lymphoma of the female breast is already rare, lymphoma of the male breast has only anecdotally been reported. Within a study of 32 lymphoma of the breast reported between 1973 and 2014 as Burkitt lymphoma, we observed a single male case, which we report here.

**Case presentation:**

A 72-years-old Caucasian man presented with a mass in his left breast. Clinical history included prior basal cell carcinoma, leiomyosarcoma, and administration of spironolactone. The reference pathology diagnosis at presentation was Burkitt lymphoma according to the Kiel Classification. The present re-investigation using fluorescence *in situ* hybridization revealed an IGH-*MY*C translocation and a break in the *BCL2* locus in the tumor cells. Thus, in light of the current WHO classification, the diagnosis was revised to high-grade B-cell lymphoma with *MYC* and *BCL2* rearrangement, Burkitt morphology (so-called “double-hit” lymphoma). Genome-wide chromosomal imbalance mapping revealed a complex pattern of aberrations in line with this diagnosis. The aberrations, including copy-number gains in chromosomes 3q and 18 and focal homozygous loss in 9p21.3, resembled typical changes of lymphomas affecting “immune-privileged” sites.

**Conclusion:**

The present case adds to the understanding of the pathogenesis of male breast lymphomas, about which hardly any molecular characterization has been published yet.

## Background

Primary lymphoma of the male breast is an extremely rare presentation affecting males in the fourth to seventh decades of life [1, 2]. To date, less than 50 cases of male breast lymphoma have been reported in the literature [1, 2]. Clinical presentation of breast lymphoma in men usually resembles the more common carcinomas with a mammographically solitary well-circumscribed painless mass in the breast and/or the ipsilateral axillary lymph nodes commonly unilateral [3–8]. Previous reports on male breast lymphomas focused mainly on the clinical and pathological features of the tumor [3–9]. Reports describing the genetic alterations of male breast lymphomas are, to the best of our knowledge, scarce. In the context of a retrospective study aiming at molecularly characterizing 32 lymphomas of the breast diagnosed historically between 1973 and 2014 as Burkitt lymphoma at the Lymph Node Registry in Kiel (Germany), we came across the case of a single male patient, which is presented here.

## Case Presentation

The tumor tissue sample of the at diagnosis 72-year-old Caucasian man was obtained at the Lymph Node Registry in Kiel (Germany) more than 25 years ago. His main complaint was an asymptomatic unilateral progressive mass of his left breast persisting over 3 months. A history of basal cell carcinoma and leiomyosarcoma was recorded 25 and 10 years prior to the lymphoma manifestation, respectively. Furthermore, long-term treatment with spironolactone was reported. A clinical examination revealed bilateral non-tender gynecomastia along with a painless swelling in his left breast and testis together with ipsilateral enlarged superficial inguinal lymph nodes. Bilateral mastectomy was performed and reference pathological analyses of the excised tissue led at that time to the diagnosis of Burkitt lymphoma of the breast according to the Kiel Classification. Neither data on treatment nor on outcome were available. During a retrospective survey of breast and ovarian lymphomas historically diagnosed as Burkitt lymphoma at the Lymph Node Registry in Kiel (Germany), archived tumor materials (formalin-fixed, paraffin embedded, [FFPE]) of the case described above were retrieved from the files and investigated applying up-to-date technologies. Use of the archived materials for molecular studies was approved by the Ethics Committee of the Faculty of Medicine, Christian-Albrechts-University of Kiel, Germany (D474/14 and D447/10). Interphase fluorescence *in situ* hybridization (FISH) studies were performed using the dual color break apart probes, LSI *MYC*, LSI IGH, LSI *BCL2* and LSI *BCL6*, as well as, the tri-color dual fusion probe LSI IGH/*MYC*/CEP8 (all probes were obtained from Vysis/Abbott Molecular, Wiesbaden, Germany). Whenever possible at least 100 nuclei were evaluated for each probe. FISH analyses were evaluated and documented using the ISIS digital image analysis version 5.0 (MetaSystems, Altussheim, Germany). For histological evaluation, tumoral tissue was stained with Hematoxylin and Eosin (H&E), as well as with a panel of monoclonal antibodies for detection of CD20, CD10, BCL2, TdT and Ki67 expression. Moreover, Epstein-Barr virus (EBV) encoded RNA (EBER) *in situ* hybridization was performed. For the analysis of genome-wide imbalances, DNA was extracted from the FFPE material with the QIAmp DNA FFPE tissue kit, (Qiagen, Hilden, Germany) and processed using the Oncoscan™ FFPE express 2.0 kit (Affymetrix, Santa Clara, CA, USA). Analyses of copy number aberrations (CNA) and copy neutral loss of heterozygosity (CNN-LOH) were performed using the TuScan algorithm of the Nexus Express for Oncoscan 3 software (Biodiscovery, El Segundo, CA, USA). Human reference genome GRCh37/hg19 was used. Gains and losses smaller than 100 Kb or encompassing less than 20 probes, as well as CNN-LOH smaller than 5000 Kb or including regions of losses were not considered.

**Fig. 1:**
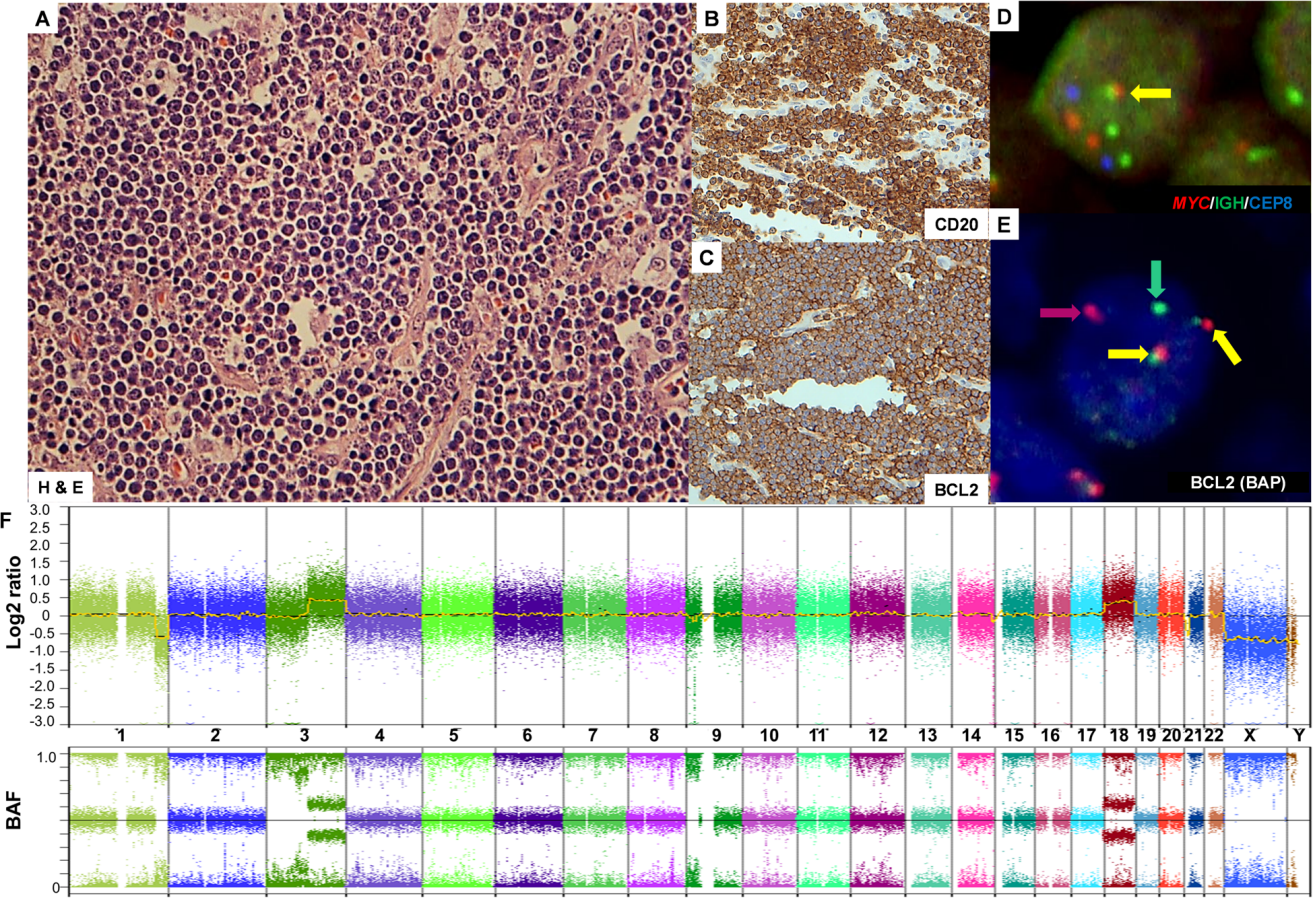
**a** Haematoxylin and Eosin (H&E) staining showing medium-sized lymphocytes with rounded nuclei and deeply basophilic cytoplasm with sporadic diffuse large cells entangling necrotic lymphocytes (magnification 40×). **b** BCL2 staining (magnification 40×). **c** CD20 staining (magnification 40×). **d-e** Interphase FISH in false-color display. (**d**) LSI *MYC* (8q24)/ IGH (14q32)/ CEP8, tri-color dual-fusion probe showing one fusion (green and red signals together, indicating by yellow arrow) signal and two red (*MYC*) and green (IGH) non-fused signals, respectively; and two blue signals of centromere 8. (**e**) LSI *BCL2* (18q21) break-apart probe (BAP) showing two co-localization signals (yellow arrows) and one isolated red and green signal (red and green arrows, respectively) **f** Genome-wide log2 ratio copy number aberration plot (top) and B-allele frequency plot (bottom)

Histopathological re-examination showed a diffuse proliferation pattern of malignant medium-sized B-lymphocytes (Fig. 1a), suggestive of mature aggressive B-cell lymphoma of Burkitt type in line with the historic diagnosis relying on the Kiel Classification. The tumor cells stained positive for CD20 and BCL2 (Fig. 1b and c) but negative for TdT, CD10 and EBER. Ki-67 showed non-representative staining most likely due to the aging effect of the stored material and thus, was considered not evaluable for technical reasons.

Molecular cytogenetic analyses using interphase FISH revealed the vast majority of cells in the tissue section to carry a chromosomal breakpoint affecting the *MYC* locus and an IGH-*MYC* fusion as well as a chromosomal breakpoint affecting the *BCL2* gene locus (Fig. 1d and e). In addition, we detected an extra signal of the non-rearranged allele suggesting a gain of the *BCL2* locus. Moreover, we observed a gain but no break of the *BCL6* locus. Based on these results and in the light of the current World Health Organization (WHO) classification of lymphoma the diagnosis was revised toward “high-grade B-cell lymphoma with *MYC* and *BCL2* rearrangements, Burkitt morphology”, commonly referred to as “double-hit lymphoma”.

Chromosomal imbalance mapping using the Oncoscan™ platform revealed copy number (CN) gains in 3q13.11-q29 and a trisomy 18 (in line with the observed FISH patterns) and CN losses in 1q41-q44, 2q31.1, 9p21.3, and 10q21.1. Moreover, CNN-LOH were detected in 3p26.3-q13.11, 9p24.3-p13.3, 15q21.1-q21.3, and 16p13.3 (Fig. 1f). Finally, attempts to perform whole-exome sequencing from the tumor unfortunately failed due to technical reasons likely caused by the limited preservation of the historic tissue.

## Discussion

Primary lymphoma of the breast is extremely rare. In contrast to female breast lymphoma that is assumed to occur in up to 0.05% of women with breast malignancy [10], presentation of the lymphoma in the male breast has been reported only sporadically in rare cases worldwide [1, 2].

In the few reported male cases, like in the patient presented here, breast lymphoma mainly affected men in the middle-to-old age groups [2–8]. Being initially considered as Burkitt lymphoma, we here rendered the diagnosis of a so-called “high-grade B-cell lymphoma with *MYC* and *BCL2* rearrangements” also called “double-hit lymphoma” with Burkitt morphology. This re-classification sets a note of caution to historic studies on the incidence and biology of Burkitt lymphomas in elderly patients or with unusual presentation.

High-grade B-cell lymphomas with *MYC* and *BCL2* and/or *BCL6* rearrangements represent a quite recently defined entity of lymphoma with aggressive nature, high genomic complexity and poor prognosis [9]. Double-hit lymphomas comprise between 32% and 78% of mature aggressive B-cell lymphoma cases with features intermediate between Burkitt lymphoma and diffuse large B-cell lymphoma [10, 11].

In line with the diagnosis of double-hit lymphoma the complexity of the genomic imbalances depicted by the Oncoscan™ array was high. The pattern of imbalances showing gains in 3q and trisomy 18 as well as homozygous loss in 9p21.3 (encompassing the region of *CDKN2A/B*) together with the lack of detectable CD10 in the presence of CD20 and BCL2 expression resembles other extra-nodal lymphoma. This holds particularly true for aggressive B-cell lymphomas at immune-privileged sites, like primary CNS lymphomas (PCNSL) or testicular lymphomas [12, 13]. The causes for this rare manifestation in the patient presented here remain unclear. Nevertheless, it is intriguing to speculate that there might be an association with the former spironolactone treatment, well known to induce gynecomastia. Alternatively, based on the clinical history of the patient with multiple neoplasia, a tumor predisposition syndrome could underly lymphoma development. Unfortunately, we could not investigate the latter hypothesis as the tumor material was of insufficient quality for whole-exome analysis.

## Conclusion

In conclusion, we described a rare EBV-negative high-grade B-cell lymphoma with *MYC* and *BCL2* rearrangements of the male breast. The similarities of the molecular findings to other types of non-nodal aggressive B-cell lymphoma affecting immune-privileged sites might indicate common pathogenetic mechanisms.

## Abbreviations

BCL2: B-cell lymphoma 2
BCL6: B-cell lymphoma 6
CD20, CD10: Cluster of differentation 20, cluster of differentiation 10 (markers of B-cell maturity)
CDKN2A/B: Cyclin-dependent kinase inhibitor 2A/B
CEP8: Chromosome enumerator probe 8 (Centromeric probe of chromosome 8)
CN: Copy number
CNA: Copy number aberration
CNN-LOH: Copy neutral loss of heterozygosity
DNA: deoxyribonucleic acid
EBER: Epstein-Barr virus-encoded RNA
EBV: Epstein-Barr virus
FFPE: Formalin-fixed, paraffin-embedded
FISH: Fluorescence *in situ* hybridization
H &E: Hematoxylin and Eosin
IGH: Immunoglobulin heavy chain
ISIS: name of Software for FISH from MetaSystem
Ki-67: Marker of cell proliferation
Kb: kilobase
LSI: Locus-specific identifier
MYC: Cellular myelocytomatosis gene
NCBI: National Center for Biotechnology Information
PCNSL: Primary central nervous system lymphoma
TdT: Terminal deoxynucleotidyl transferase
USA: United States of America
WHO: World Health Organization

## References

[1] Davies A. Double-hit lymphoma: So what? Hematol Oncol. 2019;37(1):19–23. 10.1002/hon.2581 31187528

[2] Lokesh KN, Sathyanarayanan V, Lakshmaiah KC, Suresh TM, Lokanatha D, Babu KG, *et al.* Primary breast lymphoma in males—a report of two cases with a review of the literature. Ecancermedicalscience. 2013;7:347,1–4. 10.3332/ecancer.2013.347 PMC3965189 24723970

[3] Luanow E, Kettler M, Slanetz PJ. Spectrum of disease in the male breast. AJR. 2011;196:W247–59. 10.2214/AJR.09.3994 21343472

[4] Cheah CY, Campbell BA, Seymour JF. Primary breast lymphoma. Cancer Treat Rev. 2014;40(8):900–8. 10.1016/j.ctrv.2014.05.010 24953564

[5] Corobea AB, Dumitru A, Sajin M, Poenaru R, Puşcaşu A, Chirita D, *et al.* Diffuse large b cell lymphoma in a male breast - a rare case report. Chirurgia (Bucur). 2017;112(4):477–81. 10.21614/chirurgia.112.4.477 28862126

[6] Sordi E, Cagossi K, Lazzaretti MG, Gusolfino D, Artioli F, Santacroce G, *et al.* Rare case of male breast cancer and axillary lymphoma in the same patient: a unique case report. Case Rep Med. 2011;2011:940803,1–4. 10.1155/2011/940803 PMC3191849 22007238

[7] Yim B, Park JS, Koo HR, Kim SY, Choi YY, Kim JY, *et al.* Primary breast lymphoma in an immunocompromised male patient: a case report. J Korean Soc Radiol. 2015;73(4):264–8.

[8] Zheng G, Yu H, Hemminki A, Fo A. Familial associations of male breast cancer with other cancers. Breast Cancer Res Treat. 2017;166(3):897–902. 10.1007/s10549-017-4468-1 28836035

[9] Swerdlow SH, Campo E, Pileri SA, Harris NL, Stein H, Siebert R, *et al.* The 2016 revision of the World Health Organization classification of lymphoid neoplasms. Blood. 2016;127(20):2375–90. 10.1182/blood-2016-01-643569 PMC4874220 26980727

[10] Swerdlow SH. Diagnosis of 'double hit' diffuse large B-cell lymphoma and B-cell lymphoma, unclassifiable, with features intermediate between DLBCL and Burkitt lymphoma: when and how, FISH versus IHC. Hematol Am Soc Hematol Educ Program. 2014;2014(1):90–9. 10.1182/asheducation-2014.1.90 25696840

[11] Barrans S, Crouch S, Smith A, Crouch S, Smith A, Turner K, *et al.* Rearrangement of MYC is associated with poor prognosis in patients with diffuse large B-cell lymphoma treated in the era of rituximab. J Clin Oncol. 2010;28(20):3360–5. 10.1200/JCO.2009.26.3947 20498406

[12] Batlevi C, Rapaport F, Wang L, Intlekofer AM, Copeland AR, Jungbluth AA, *et al.* Distinctive genomic alterations in testicular diffuse large b cell lymphoma. Blood. 2015;126(23):3655.

[13] Chapuy B, Roemer MGM, Stewart C, Tan Y, Abo RP, Zhang L, *et al.* Targetable genetic features of primary testicular and primary central nervous system lymphomas. Blood. 2016;127(7):869–81. 10.1182/blood-2015-10-673236 PMC4760091 26702065

